# Optical Filters Based on Cholesteric, Blue and Sphere Mesophases

**DOI:** 10.3390/polym14224898

**Published:** 2022-11-13

**Authors:** Changli Sun, Jiangang Lu

**Affiliations:** National Engineering Lab for TFT-LCD Materials and Technologies, School of Electronic Information and Electrical Engineering, Shanghai Jiao Tong University, Shanghai 200240, China

**Keywords:** twist-structure liquid crystals, filter, photonic properties, templating technology

## Abstract

An optical filter is one of the indispensable devices in massive and high-speed communication, optical signal processing, and display. Twist-structure liquid crystals, cholesteric liquid crystals, blue-phase liquid crystals, and sphere-phase liquid crystals show potential application in optical filters originating from the periodic nanostructures. Wavelength and bandwidth tuning can be controlled via temperature, electric fields, light, angle, spatial control, and templating technology. In this review, we discuss the recent developments of twist-structure liquid crystal filters.

## 1. Introduction

Wavelength-selective and band-pass filters are fundamental and essential devices in optical communication for satisfying the acute need for massive and high-speed communication. They are the essential components of optical wavelength-division multiplexing and orthogonal frequency-division multiplexing systems in optical fiber communication [1,2,3], visible light communication [4,5], and microwave communication [6]. Liquid crystals (LCs) are promising materials in the optical communication field due to the advantages of low driving power, low power consumption, high birefringence, and large electro–optic effect [7,8,9]. LC is a state of matter between isotropic liquid and solid phases, possessing both the fluidic characteristics of liquid and the order properties of crystals [10,11]. The introduction of chirality to the LC system has an important impact on the properties [11]. Twist-structure liquid crystals (TSLCs) are a class of variant LCs with twisted LC molecules, consisting of blue-phase LCs (BPLCs), cholesteric LCs (CLCs), and sphere-phase LCs (SPLCs) [12]. TSLCs show a good application in optical communication devices, such as wavelength-selective filters, optical attenuators, optical switches, and beam steerers [13,14,15]. The wavelength range of TSLCs can be tuned by doping different concentrations of chiral dopant, forming spatial gradients, designing device structure, applying temperature, or irradiating the material with ultraviolet light [16,17,18,19], which makes them attractive in tunable optical filters [20,21,22,23]. Gao et al. reported the development of templated TSLCs and summarized the potential photonic applications, including lasing, optical filters, grating, etc. In this work, we focus on optical filters based on TSLCs, promising stimuli-responsive materials for wavelength and bandwidth tuning. We introduce recent advances in TSLC filters tuned by temperature, electricity, light irradiation, incident angle, spatial control, and templating technology. We demonstrate the basics and photonic properties of TSLCs before discussing TSLC filters in detail.

## 2. Photonic Properties of TSLCs

LC is a state of matter between an isotropic liquid state and a crystalline solid state, possessing the fluidity properties of a liquid and the order characteristics of a crystal [10,11]. Molecules of nematic LCs tend to orient in one direction, considered to be the LC molecular director. The introduction of chirality to nematic LCs, which promotes the formation of TSLCs, has an important influence on the properties [11]. The twisting power between the TSLC molecules can induce several phases of different structures, including CLCs with helical superstructures, BPLCs with three-dimensional self-organized structures, and SPLCs with three-dimensional twist-structures [24].

**Figure 1:**
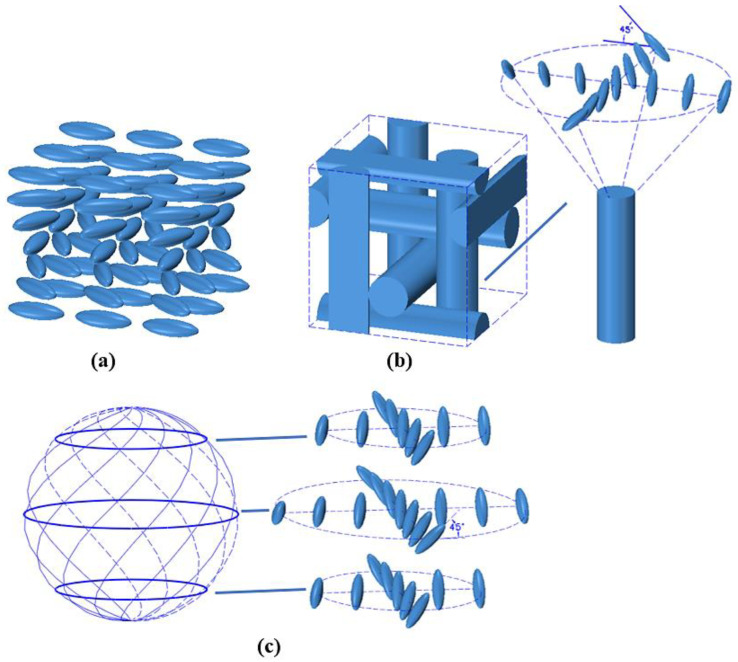
Organization of molecules in (**a**) CLC (planar texture), (**b**) BPLC (BP I), and (**c**) SPLC.

CLCs are composed of molecules arranged into a helical twist structure, and the molecules align perpendicularly to the twist axis [25,26]. CLCs exhibit two stable states at the initial state: the focal conic state and the planar state. In a focal conic texture, the helical axes are randomly arranged, leading to strong light scattering. The planar texture exhibits strong selective Bragg reflection due to the uniform orientation of the molecules (Figure 1a) [27]. It reflects circularly polarized light with the same handedness as the CLCs helix [28]. Outside the reflection band, both the right- and left-circularly polarized light are transmitted [29]. The selective Bragg reflection’s peak wavelength λ_P_ can be given by $\lambda_{P}=\bar{n}\times P\times cos\theta$, where $\bar{n}$ is the average refractive index, P is the pitch, and θ is the angle of incident light [10,30]. At normal incidence, θ is equal to 0°. CLCs usually show a blue/red shift on deviating from the normal incidence [31]. The pitch also contributes to the spectral position of the reflection wavelength, which can be determined by $P=\frac{1}{\left[c\right]\times HTP}$, where [c] is the concentration and HTP is the helical twisting power of the chiral dopant [10]. The bandwidth of the reflection spectrum Δλ can be expressed as $\Delta \lambda =\left(n_{e}-n_{o}\right)P=\Delta nP$, where Δn is the birefringence [29]. The HTP of a chiral dopant is indicative of its ability to induce twist structures in nematic LCs [10,32]. The pitch of CLC mixtures can be controlled using temperature, light, and electric fields. By mixing CLC with reactive polymers and photopolymerization in the presence of a photo-initiator, a polymer-stabilized CLC can be obtained. An anchoring effect imposed by the polymer network exists in CLCs [29]. Refilling liquid crystals into the polymer template after the washout process can form LC templates with helical structures [12]. Polymer-stabilized CLC templates have several advantages, such as an increase in stability, an enhancement in reflectivity, the possibility of multiple reflection bands, and a flexibly changeable reflection band featured by refilling different materials.

BPLCs with two-dimensional twist structures usually exist in narrow temperature ranges between the isotropic phase (Iso) and chiral nematic phase (N*) [33,34,35]. As temperature decreases, BP molecules tend to self-organize into three kinds of phases: BP III (unknown, maybe an amorphous network of disclination lines or a quasi-crystal), BP II (simple cubic symmetry), and BP I (body-centered cubic symmetry) (Figure 1b) [36,37]. BP II and BP I cubic structures have been confirmed by Kossel diffraction line analysis and small-angle X-ray scattering [38,39]. Their periodic lattice structures give rise to many remarkable properties, such as Bragg reflection, Kerr effect, scattering, and optical rotation [12,40]. Double-twist cylinders and defects coexist in the BPLC lattice, resulting in a relatively high free energy and narrow temperature range. To solve this problem, polymers are used to stabilize the lattice superstructures, supporting the cubic structure by being inserted into the BP disclination lines [33]. Polymer-stabilized BPLCs show several intrinsic features, including optically isotropic status, sub-millisecond response time, and a periodic three-dimensional helical structure with a periodicity on the scale of several hundred nanometers [41]. Polymer-stabilized BPLCs can be triggered by external stimuli that result in structural changes, such as temperature, light, electricity, humidity, and force, resulting in the shift of the Bragg reflection wavelength and variation in the bandwidth [42,43,44,45,46]. According to Bragg’s law, the selective reflection wavelength (*λ_c_*) can be correlated with the crystal properties by $\lambda_{c}=2nd_{hkl}$, where $n=\sqrt{\left(n_{e}^{2}+2n_{o}^{2}\right)/3}$ is the isotropic refractive index, *n_e_* and *n_o_* are the extraordinary and ordinary indices of refraction, respectively, *d_hkl_* represents the periodicity along the [*hkl*] axis, and *h*, *k*, *l* are the Miller indices. For the initial cubic state, the central wavelength (*λ*_0_) related to the lattice constant *a*_0_ can be expressed as $\lambda_{0}=2nd_{0}=2na_{0}/\sqrt{h^{2}+k^{2}+l^{2}}$, where *d*_0_ is *d_hkl_* in the cubic state [47]. The Bragg reflection bandwidth Δλ can be given by Δλ=ΔnP, where the birefringence is $\Delta n=n_{e}-n_{o}$ [27]. Polymer-stabilized BPLCs show a Kerr response to electric fields [48]. In the voltage-off state, the BPLC is optically isotropic, while at the voltage-on state, the birefringence of BPLC is induced by the electric field. This inducing process under electric field is defined as the electric-field-induced birefringence effect. The induced birefringence (*Δn_ind_*(*E*)) can be demonstrated by the extended Kerr equation [49,50]: ${\left(\Delta n\right)}_{ind}=\lambda KE^{2}={\left(\Delta n\right)}_{s}\left(1-\exp\left[-{\left(\frac{E}{E_{s}}\right)}^{2}\right]\right)$, where *λ* is the wavelength of the incident light, *K* represents the Kerr constant, $\Delta n_{s}$ is the saturated induced birefringence, *E* represents the intensity of the applied electric field, and *E*_*s*_ is the intensity of the saturated electric field. The ordinary refractive index (*n*_o_(*E*)) depending on *E* can be expressed as $n_{o}\left(E\right)=n_{iso}-{\left(\Delta n\right)}_{ind}\left(E\right)/3$, where *n_iso_* is the refractive index in initial state [50,51,52,53].

An SPLC consists of self-organized nanoscale periodic three-dimensional twist structures (3-DTSs) induced by a chiral dopant, usually observed in a narrow temperature range between the isotropic and blue phase or isotropic and chiral nematic phase [54,55,56]. 3-DTSs are composed of several planar layers of double-twist LC molecules (Figure 1c). The twisted angle of the LC molecules on the outermost circumferences gradually decreases from the equatorial plane to the poles. Disclinations among 3-DTSs and 3-DTSs coexist in SP, leading to weak stability and fast response. An SPLC has the characteristics of a low driving voltage, fast switching time, and light scattering. Its theoretical mechanism for filtering still requires further investigation.

## 3. Filters Based on TSLCs and Templated-TSLCs

TSLC filters can be triggered by several stimuli to generate structural change, resulting in a shift of the Bragg reflection wavelength and variation in the bandwidth. The effects of templating technology, temperature, electricity, light irradiation, incident angle, and spatial control on TSLC filters are presented below.

### 3.1. Templating Technology

Templating is one approach that transfers the features of a host medium into a guest matrix through a set of chemical and physical processes. It is a replication of fundamental features under structural inversion [57]. A polymer template can be prepared by photopolymerizing LC pre-polymers and then washing out the remaining molecule mixtures [58,59]. A variety of LCs, including nematic LCs, chiral nematic LCs, and pre-polymers, are candidates for materials refilled into polymer templates [60]. Filters based on templating technology have the advantages of high reflectivity, multiple reflection peaks, and a flexibly changeable photonic band gap (PBG) [27,61].

**Figure 2:**
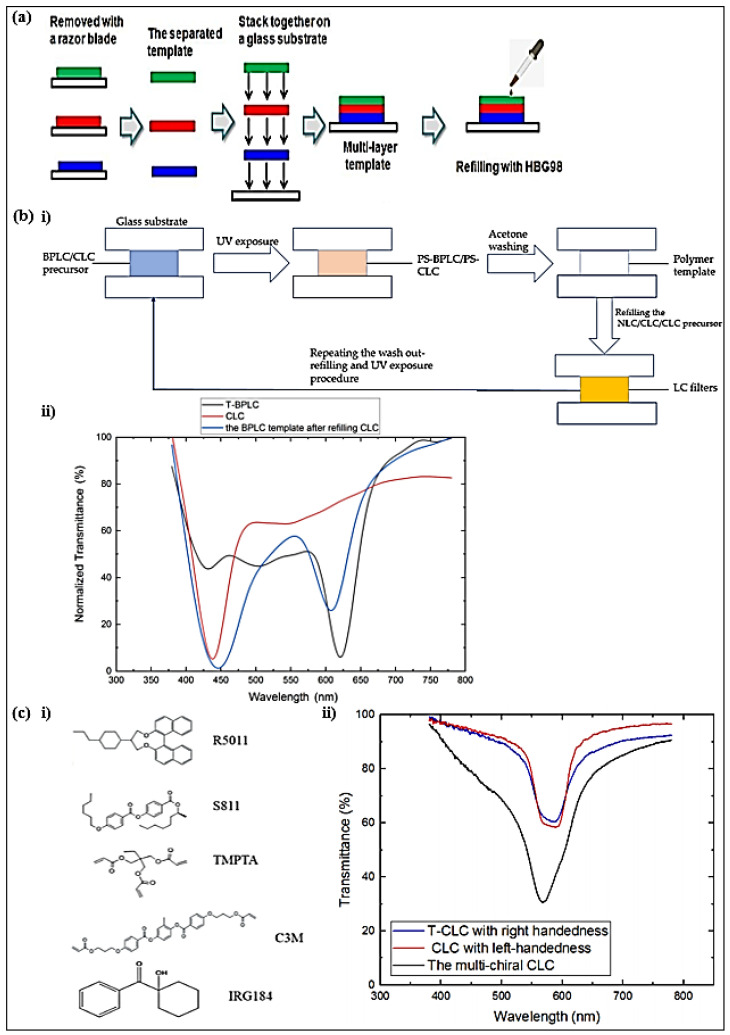
(**a**) The fabrication process of the multi-layer templated BPLC filter. Reproduced with permission from Ref. [62]. MDPI, 2019. (**bi**) Flow chart of multiple refilling process. (**bii**) Transmittance spectra of templated-BPLC, CLC, and dual-wavelength LC filter. Reproduced with permission from Ref. [63]. MDPI, 2021. (**ci**) Materials used in experiments. (**cii**) Transmission spectra of multi-chiral CLC filters with single layer. Reproduced with permission from Ref. [64]. MDPI, 2021.

A multi-layer templated BPLC filter reflecting multi-wavelength without intermediate dielectric layers was fabricated (Figure 2a) [62]. To obtain the template, the glass substrates of the cell were separated, and the polymer-stabilized BPLC film was placed into ethanol to wash out the residual LC, chiral dopant, nonreactive monomers, and the photo-initiator. After laminating the templates of different reflection wavelengths and refilling nematic LCs into the multi-layer template, the BPLCs were reconstructed. The templated-BPLC filter showed a narrow reflection bandwidth (<15 nm), good angular stability, and stable reflection with a temperature shift.

Compared to the LC filter with a multi-layer structure, a single-layer structure LC filter could significantly simplify the device structure and streamline the fabrication process. Hence, a multi-wavelength TSLC filter and a bandwidth-tunable CLC filter of a single-layer structure were implemented with a multiple wash-out–refilling process [63]. By refilling a CLC with a different pitch from that of the target template into a BPLC template, a single-layer LC filter with multi-reflection peaks was obtained (Figure 2b). By refilling a CLC template with CLCs of adjacent pitch sequentially, a bandwidth-tunable single-layer filter could be realized. The FWHM of the bandwidth-scalable CLC filter could be continuously broadened by 96% when compared with that of the original filter.

To improve the maximum reflectance of a single-layer CLC filter, a high-reflectivity CLC filter reflecting both right- and left-circularly polarized light was proposed [64]. A filter with hyper-reflectivity was obtained by refilling a left-handed (LH) CLC into a right-handed (RH) CLC template (Figure 2c). The RH polymer-stabilized CLC precursors consisted of BPH006, R5011, C3M, TMPTA, and IRG184. The refilling LH CLC mixtures comprised BPH006 and S811. The hyper-reflectivity was related to the wavelength consistency. Different from the single-handed LC filter, the multi-chiral LC filter showed hyper-reflectivity due to the coexistence of right- and left-handedness.

A single-layer LC filter, multi-wavelength LC filter, multi-phase LC filter, and multi-chiral LC filter could be realized using the templating technology. The TSLC filter with templating technology featured high flexibility, high reflectivity, a wide tunable range, and good stability. The handedness of the template, the phase of the refilling LCs, and the wash-out–refill process were important factors for achieving a TSLC filter based on the templating technique.

### 3.2. Temperature Variation

The reflection bands of the filters associated with the helical pitch, order parameter, and refractive indices are related to temperature due to the thermodynamic behavior of the LC molecules [65,66,67]. The temperature-dependent characteristics of the LC filters cover the central wavelength and Bragg reflection bandwidth. For BPLCs, the temperature dependence of the Kerr constant, which is related to the induced birefringence and pitch length, is of great relevance and of fundamental importance [68,69].

**Figure 3:**
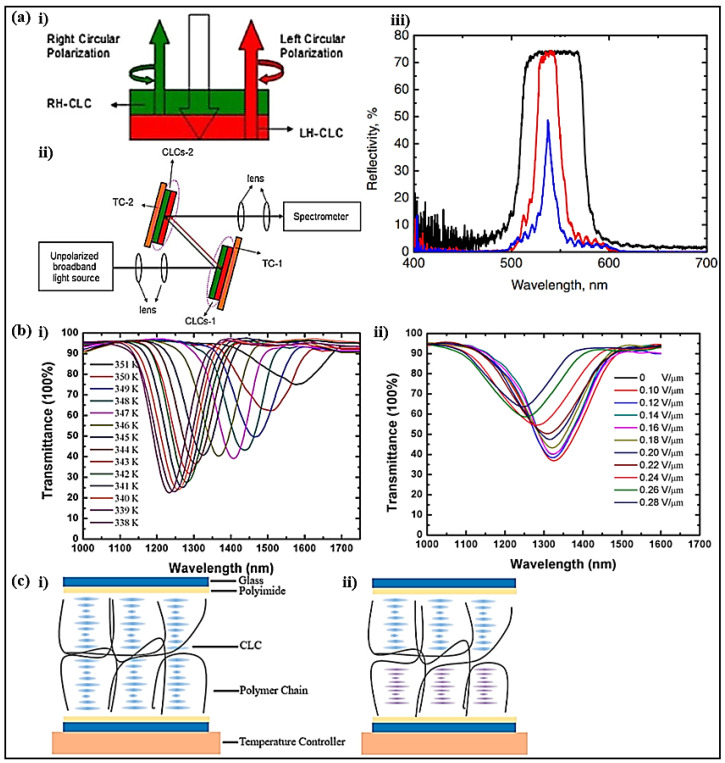
(**ai**) Combination of RH-CLC and LH-CLC. (**aii**) Schematic diagram of the experiment setup. (**aiii**) Reflection spectra of the filter. Reproduced with permission from Ref. [70]. Copyright 2014 The Japan Society of Applied Physics. (**b**) Transmission spectra of the filter versus (**bi**) temperature and (**bii**) electric field. Reproduced with permission from Ref. [71]. MDPI, 2019. (**c**) The mechanism illustration of the holding treatment (**ci**,**cii**). Reproduced with permission from Ref. [72]. MDPI, 2019.

In order to improve the reflectivity, a polarization-independent tunable optical filter combining LH and RH CLCs as a unit was demonstrated (Figure 3a) [70]. The bandwidth of the reflection band decreased as the reflection band of CLC-1 red-shifted with decreasing temperature and that of CLC-2 blue-shifted with increasing temperature. The bandwidth of the high reflectivity CLC filter could be adjusted from 10 to 70 nm, and the central wavelength could vary from 573 to 500 nm with the temperature ranging from 23 to 50 °C.

In addition to the CLC filter, a near-infrared SPLC filter with a low operating electric field and large temperature gradient was proposed (Figure 3b) [71]. During the cooling process from the sphere phase to N*, the structure varied from a 3-DTS to a helical twist structure. Due to the sensitivity of the 3-DTSs to external stimuli, a central wavelength tuning range from 1580 nm to 1324 nm with a temperature gradient of 42.7 nm/K was obtained. In addition, an electrical central wavelength adjustment of over 76 nm with an operating electric field of 0.3 V/µm was realized.

Considering the effect of temperature on the LC filter, the performance of the sectional polymerization process on the tunable TSLC filters was demonstrated [72]. As the temperature decreased rapidly, the pitch of TSLCs at the bottom close to the temperature controller was shortened owing to the helical-twisting power variation, while that at the top remained due to the long distance from the temperature controller (Figure 3c). The reflection bandwidth of the CLC filter and the BPLC filter could be widened by the holding treatment from 120 nm to 220 nm and from 45 nm to 140 nm, respectively.

The tuning of the central wavelength and the bandwidth of the TSLCs was based on the temperature-dependent pitch variation, refractive indices change, and the reorientation of the LC molecules. Several factors influencing the reflection band had a strong relationship with temperature, including the helical-twisting power of the chiral dopants, elastic constants, Kerr constant, viscosity, and the order parameters of the LCs. The temperature responses of the TSLCs were critical for their application in filters.

### 3.3. Electric Field Modulation

Among various stimuli, the electric field shows good feasibility and high efficiency in inducing the reorientation of the LC molecules [73]. For BPLCs, three typical and progressive effects of the electric field are known, including a local reorientation of the LC director, a distortion of the cubic lattice, and a phase transition to lower ordered phases [74]. The reflection bandwidth of the polymer-stabilized CLCs with negative dielectric anisotropy can be changed by direct current (DC) electric fields due to the absorption of cations by the polymer network [75].

**Figure 4:**
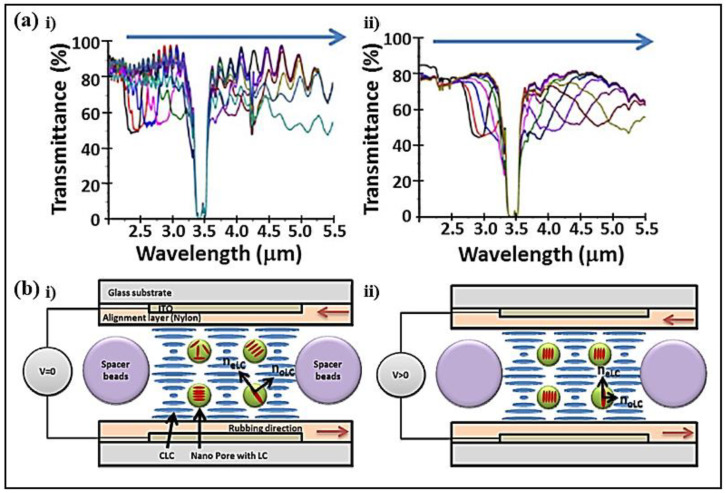
(**a**) Red-shifting tuning of polymer-stabilized CLC cells with (**ai**) silver nanowires and (**aii**) graphene electrodes. Reproduced with permission from Ref. [76]. Copyright 2016 Optical Society of America. (**b**) Scheme of the proposed polymer-stabilized CLCs with electric field (**bi**,**bii**). Reproduced with permission from Ref. [77]. Copyright 2014 Optical Society of America.

The electrical tuning of the central wavelength and the bandwidth of the CLC bandpass filters in the infrared (3-5 μm) was reported [76]. The substrates coated with silver nanowires and graphene mid-wave infrared (MWIR) transparent electrodes were fabricated (Figure 4a). Under a DC field of 110 V, the central wavelength of the filter eventually reached 4.90 μm in the MWIR band. With a voltage ranging from 0 to 20 V, the reflection band was broadened and extended to cover a wavelength range of 2500–4200 nm, obtaining a bandwidth of nearly 2000 nm.

A wavelength shift of 141 nm was realized by electric control on a partly polymerized chiral LC [77]. The LC filter consisted of a mixture of photopolymerizable LC, non-reactive nematic LC MDA, and a chiral dopant (Figure 4b). Upon applying an electric field, the ordinary refractive index of the LC contributed, and the refractive indices of the mixture decreased. Since the pitch was maintained by the polymer template, the photonic band edges both programmed a blue shift. The filter featured high reflectivity over the reflection band and possessed a switching-on time of 50 µs and a switching-off time of 20 µs.

**Figure 5:**
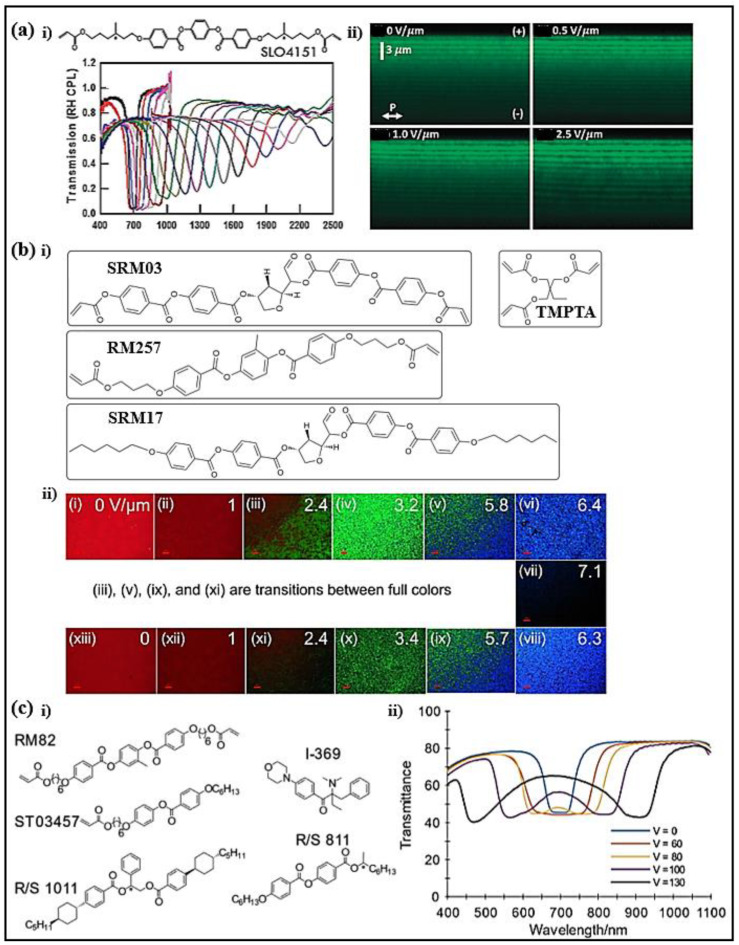
(**a**) Transmission spectra and cross-sectional images of the polymer-stabilized CLCs. Reproduced with permission from Ref. [78]. Copyright The Royal Society of Chemistry 2015. (**bi**) Molecular structures of materials. (**bii**) Electric-field-dependent reflection spectra. Reproduced with permission from Ref. [79]. NPG Asia Materials, 2020. (**ci**) Chemical structures of materials. (**cii**) Transmittance spectra for bandwidth splitting. Reproduced with permission from Ref. [80]. Copyright 2020 WILEY-VCH.

Polymer-stabilized CLCs with negative dielectric anisotropy featuring a large magnitude (exceeding 1500 nm) and invertible tuning under a DC field were reported (Figure 5a) [78]. The optical response and relaxation behavior upon the application of a DC electric field were strongly related to the viscoelastic properties of the polymer network. The nonlinear distortion of the pitch and electromechanical response of the polymer network contributed to the reflection variation.

Dynamic control of the reflection band in the monodomain polymer-stabilized BPLCs upon an electric stimulus was demonstrated [79]. Compared with achiral monomers, chiral monomers contributed to the stability of the double-twist cylinders due to the intrinsic chiral structure. The internal structure of the mixture could be well maintained even under a strong electric field since the polymer network was formed in both disclination lines and bulk LCs (Figure 5b). The spectral range reached ~216 nm by adjusting the chiral monomer and LC concentrations in the mixture. Via the application of an electric field, the total reflection tuning range could be 241 nm, and the switching time was <350 ms.

A new electro–optic phenomenon that a single selective reflection splits into two distinct reflections under an electric field was observed in the CLC phase [80]. The distinctive phenomena originated from the introduction of side-chain liquid crystalline mesogens and were dependent on the compositions of the mixtures (Figure 5c). The distinguished control on two CLC elements was demonstrated. The CLCs showed functional opportunities in several devices, such as spectrum-variant polarization filters.

The electric field could change the refractive indices of the LCs with dielectric anisotropy by tilting the LC molecules, resulting in the shift of the reflection band. The variation in the reflection band induced by the electric field included reflection-band splitting, red shift, and left shift. The value, direction, frequency, and mode of the electric field might have an influence on filter performance.

### 3.4. Light Control

Light control is a preferred external stimulus for LCs due to the advantages of remote, temporal, and spatial manipulation [81,82]. In such LC systems, photoresponsive chiral switches, mainly composed of chiral centers and photoswitches, are generally doped into nematic LC materials [82]. Photoswitches undergo configurational changes upon photoisomerization, such as the reversible trans-cis isomerization of azobenzenes and menthone derivatives [83,84]. The isomerization leads to the variation in the helical twisting power, the pitch length, and, consequently, the selective reflection of the LCs [85].

**Figure 6:**
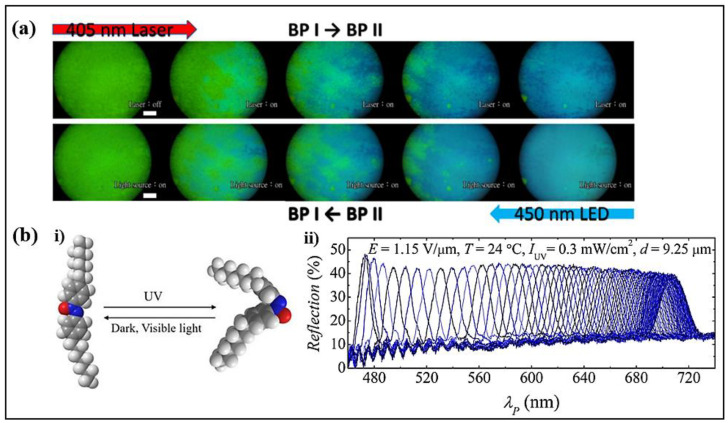
(**a**) Polarizing optical microscopic images of the aligned chiral–azobenzene-doped BPLCs sample during the illumination of different light sources. Reproduced with permission from Ref. [86]. MDPI, 2020. (**bi**) Trans and cis isomers of D7AOB molecule. (**bii**) Bragg reflection spectra of the Ch_OH_ cell. Reproduced with permission from Ref. [25]. Copyright 2021 American Physical Society.

The properties of photoresponsive self-assembled chiral–azobenzene-doped BPLCs were investigated [86]. During the cooling process, surface alignment could contribute to the induction of more uniform and diverse BP structures, containing BP II, BP I and BPS-like phases (Figure 6a). The photoisomerizations of trans→cis and cis→trans occurred when chiral–azobenzene-doped dopants were irradiated by light with wavelengths of 405 and 450 nm, respectively. The photoisomerization process contributed to isothermal phase transition and reflection-band shifting, improving the possibility of all-optical control on BPLCs.

Electric and photo control on an oblique helicoidal cholesteric (Ch_OH_) LC doped with an azoxybenzene derivative were investigated [25]. Light irradiation caused trans-cis photoisomerization of azoxybenzene dopants, leading to variations in the pitch and diffractive properties of Ch_OH_ (Figure 6b). The maximum central wavelength shift of the Bragg reflection reached about 235 nm by the photoisomerization of 5 wt% achiral molecules, indicating the ultra-sensitivity of Ch_OH_ to light irradiation.

Azobenzenes and menthone derivatives doped LCs underwent isomerization with light control, resulting in changes in the twist structure, pitch, and birefringence. Photo-induced manipulation had the advantages of remote control, fast response time, and ease of addressability. The factors affecting filter performance contained the light irradiation time, the wavelength of the light stimuli, and the intensity of the light irradiation.

### 3.5. Angle Change

The selective reflection of BPLCs relates to the periodicity of the cubic lattice. The Bragg reflection band shifts with the variation in viewing angle [87]. The first-order reflection of a monodomain BPLC experiences a blue shift, while the second-order reflection undergoes a red shift with an increase in incident angle [88]. For the planar state of CLCs, the Bragg reflection spectrum closely correlates with the incident angle of the light [89]. The reflection intensity decreases as the detection angle gradually deviates away from the Bragg reflection angle [90].

**Figure 7:**
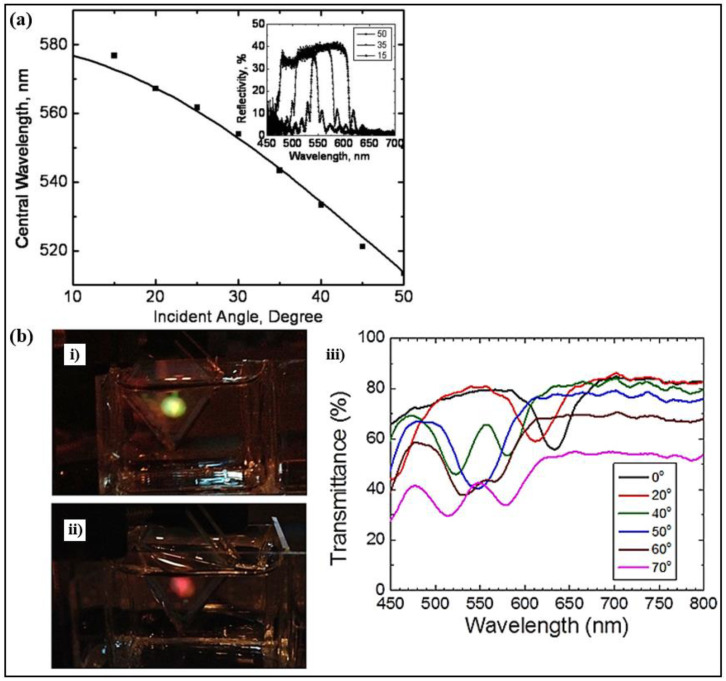
(**a**) Central wavelength of tunable filter versus incident angle. Reproduced with permission from Ref. [91]. Copyright 2011 Optical Society of America. (**bi**) First-order and (**bii**) second-order reflection of the BPLC cell. (**biii**) Transmission spectra of polymer-stabilized BPLC. Reproduced with permission from Ref. [88]. Copyright 2013 AIP.

An optical filter consisting of two CLCs in reflection mode was demonstrated. The filter featured a tunable wavelength and a variable bandwidth [91]. A wavelength-tuning range of 574 nm–527 nm was achieved by rotating the angle of two CLCs together from 15° to 50° (Figure 7a). Additionally, a bandwidth variation of 80 nm–10 nm could be obtained by changing the relative angle between two CLCs from 0° to 35°. The central wavelength and bandwidth could be adjusted to the desired value according to the requirement.

The properties of a monodomain BPLC in angle-dependent reflection were investigated [88]. Different from a CLC with a helical structure, the monodomain BPLC presented diverse reflection orders at different crystal planes. With an increase in incident angle, the first-order reflection experienced a blue shift, while the second-order reflection underwent a red shift (Figure 7b). The characteristics of monodomain BPLC photonic devices with oblique incidence would be affected by the angular dependency properties.

The properties of the Bragg reflection band, covering the central wavelength and bandwidth, were associated with the incident angle of the light. The influencing factors on the filter characteristics included the value of the incident angle, the value of the viewing angle, the incident planes, and the orientation of the crystal planes. Reflections orders might be different for various incident angles.

### 3.6. Spatial Design

Spatial tuning is one appropriate method enabling good maintenance, high stability, and the wide tunable range of LC devices after polymer stabilization [92,93]. Along the space variation direction, a continuous linear change of helical pitch takes place [94,95]. The continuous change originates from the match between the pitch gradient concentration and the helical pitch determined by the space. Hence, the central wavelength of PBG spatially changes due to the pitch variation along the direction of LC cell [96].

**Figure 8:**
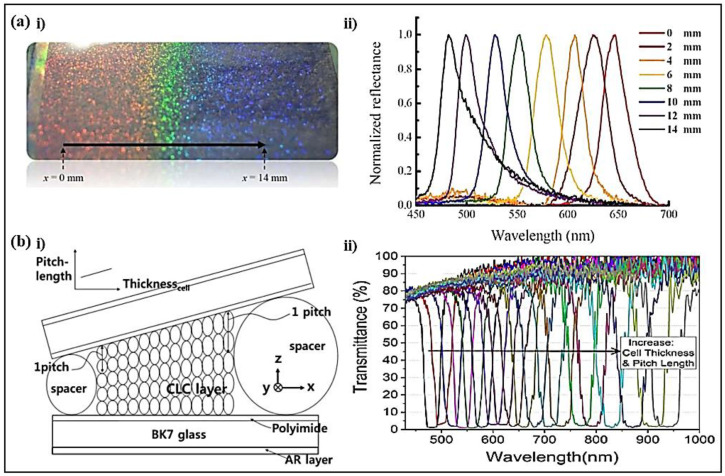
(**ai**) Photograph of the gradient-pitched BPLC. (**aii**) Reflection spectra of the sample. Reproduced with permission from Ref. [92]. Copyright 2016 Nature Publishing Group. (**bi**) Schematic diagram of the wedge CLC cell. (**bii**) Spectra of the tunable filter. Reproduced with permission from Ref. [96]. Copyright 2018 IEEE.

A polymer-stabilized BPLC with a large pitch gradient and a wide spatially tunable band covering the visible region was developed (Figure 8a) [92]. The cell was fabricated by injecting two BPLC mixtures with different chiral concentrations in reverse directions. The mixtures diffused in the cell and a pitch gradient formed. The spectral tuning range reached 165 nm covering nearly the entire visible region. The polymer-stabilized BPLC filter showed the advantages of wide tunability, high reliability, and no extra controlling sources.

A central wavelength-tunable and bandwidth variable optical filter composed of four LH and RH CLCs without extra components was reported [96]. The filter consisted of CLC wedge cells with a lateral continuous pitch gradient (Figure 8b). The central wavelength of the reflection band could be spatially adjusted from 470 nm to 1000 nm. The bandwidth underwent a reversible change from 60 nm to 18 nm. The polarization state of the band pass filter could be made via cell alignment.

The central wavelength and bandwidth of TSLCs could be spatially tuned due to the pitch gradient distribution in the cell. Filters based on spatial control had the advantages of wide band, high stability, and continuous tuning. The elements affecting the reflection band covered the varying chiral concentrations along the spatial position, the cell thickness, and the refractive indices of LCs.

## 4. Conclusions

TSLCs show great potential application in optical filters due to low power consumption, relatively low cost, diverse actuation modes, and simple fabrication process. Among TSLCs, CLCs, BPLCs, and SPLCs dominate the current literature. CLCs exhibit selective reflection of incident radiation due to the periodic helical structure. The pitch in the sub-micrometer range of CLCs enables great potential application in mid-wave infrared filters. Methods for realizing an accurate control of the wavelength and bandwidth of CLCs in the infrared region are being investigated. BPLCs show structural color caused by the 3D periodic nanostructure. Optically isotropic, no need for polarizers, alignment-layer free, and sub-millisecond response time are the advantages. However, the high driving voltage and hysteresis of BPLCs are still challenges. SPLCs reveal a reflection band originating from the periodic 3-DTSs. Its sensitivity to external stimuli might contribute to the fast switching time and low driving voltage. The filtering properties of SPLCs still need further investigation. The Bragg reflection of TSLCs promotes their application in filters. The effects of triggers on these TSLC filters, such as temperature, light, and electricity, are discussed in this review. The factors that affect the tuning of TSLC filters, including templating technology, temperature, electricity, light irradiation, incident angle, and spatial control, are presented.

## Data Availability

Our study did not report any data.
